# Spot diagnosis: An ominous rash in a newborn

**DOI:** 10.1186/1824-7288-35-10

**Published:** 2009-04-30

**Authors:** Kam-Lun Hon, King-Woon So, William Wong, Kam-Lau Cheung

**Affiliations:** 1 Department of Paediatrics, The Chinese University of Hong Kong, Prince of Wales Hospital, Shatin, Hong Kong

## Abstract

Purpura fulminans (PF) is an ominous cutaneous condition usually associated with meningococcemia. PF in the newborn is rarely reported. We report the case of a female preterm infant with extensive PF due to group B streptococcus (GBS) septicemia. She developed multi-organ system failure despite neonatal intensive care support and succumbed 9 days later. GBS, sensitive to penicillin, was isolated from the blood cultures of the mother and the infant. Invasive early GBS infection is common in the newborn and is empirically treated with prompt institution of intravenous antibiotics. PF associated with GBS is a rare cutaneous sign that must not be missed. Mortality remains high despite aggressive treatment and ICU support.

## Introduction

Purpura fulminans (PF) is an ominous cutaneous condition usually associated with meningococcemia [1-9]. PF in the newborn is rarely reported [6-12] We report the case of a female preterm infant with extensive PF due to group B streptococcus (GBS) septicemia and discussion issues of management of this rare but often fatal condition.

## Case

Purupura fulminans (PF) was immediately evident in a moribund 2.7 kg newborn girl delivered by emergency caesarean section for fetal tachycardia (200/minute by cardiotocography) at 35 week gestation 1. There was no family history of bleeding disorder. The membranes were ruptured 3 hours prior to delivery. The mother developed intrapartum fever (38.9°C) with chills and rigors and was given intravenous ampicillin and gentamicin 23 minutes before delivery by emergency caesarean section. At birth, the baby was apneic with heart rate of 80/minute. She cried and the heart rate responded upon bag and mask ventilation for 1 minute. Apgars were 8 and 10 at 1 and 5 minutes, respectively. On arrival at the NICU, the baby developed further apneas with cyanosis followed by tachypnea, insucking chest and grunting. Her mean arterial blood pressure was 30 mmHg and heart rate 190/minute. Arterial blood gas analysis showed a pH of 7.19, pCO_2 _8.03 kPa, pO_2 _2.25 kPa, and base excess of -6.9 mmol/L. Respiratory support (nasal continuous positive airway pressure of 5 cm H_2_O with 8 L/min of oxygen), normal saline bolus, and intravenous penicillin plus gentamicin were administered within the first hour of resuscitation. In the next 2 hours, she remained hypotensive despite further saline boluses, dopamine infusion and mechanical ventilation. Group B streptococcus, sensitive to penicillin, was isolated from the blood cultures of the mother and the infant. She was aggressively treated with broad antibiotic coverage, cardiopulmonary support with mechanical ventilation and multiple inotropes, and peritoneal dialysis (Table 1). The purpuric rash became more extensive and she developed progressive multi-organ system failure despite full intensive care support and succumbed 9 days later

**Figure 1:**
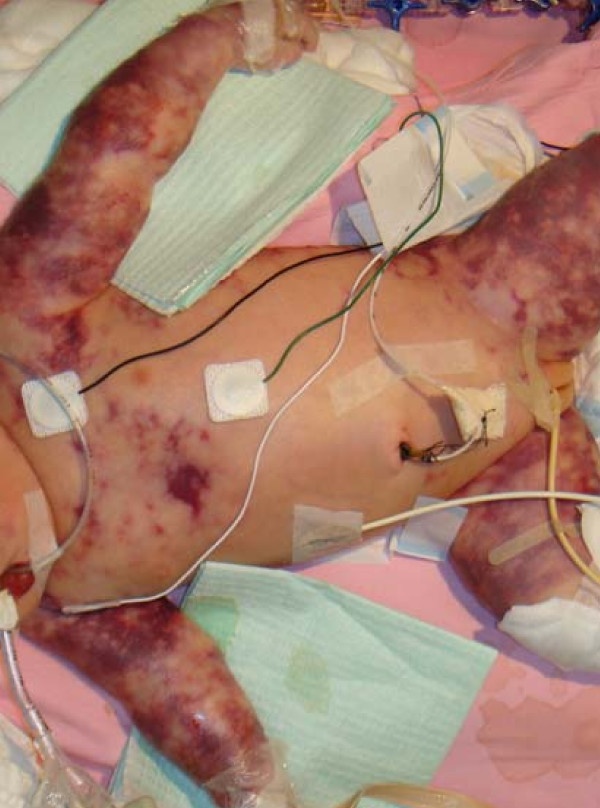
**Purupura fulminans (PF)** was immediately evident in a moribund 2.7 kg newborn girl delivered by emergency caesarean section for fetal tachycardia (200/minute by cardiotocography) at 35 week gestation.

**Table 1:** Multi-organ system failure in the neonate with group B streptococcal septicemia

**Organ system**	**Abnormal findings**	**Management**
Cardiovascular	Cardiogenic and distributive shock; poor perfusion; ejection fraction 54% and fractional shortening 26% . Highest creatine phosphokinase 1033 U/l and cardiac troponin 0.45 ug/l	Intravenous saline boluses, dopamine, dobutamine, epinephrine, hydrocortisone, milrinone, vasopressin
Respiratory	Respiratory failure with hypercarbnia and diffuse haziness on chest radiograph	Mechanical ventilation, FiO_2 _1.0, surfactant, vecuronium
Renal	Passed urine at 10 hours of life; persistent oliguria; anuria 4 days later. Highest creatinine 153 umol/l	Intravenous frusemide; peritoneal dialysis; gentamicin stopped
Septicemic	Group B streptococcus, sensitive to penicillin, isolated on surface swabs, and in baby and mother's blood cultures; highest C-reactive protein 12.9 mg/l	Intravenous penicillin and gentamicin initially; ampicillin; cefotaxime; meropenim and vancomycin empirically; intravenous immunoglobulin Subsequently on high-dose penicillin and cefotaxime when group B streptococcus and sensitivity were available.
Hematologic	Disseminated intravascular coagulopathy with lowest hemoglobin 8.6 g/dl, thrombocytopenia 13 × 109/l, D-dimer 9735 ng/ml, prothrombin time 60 seconds, and activated plasma thromboplastin time 120 seconds	Packed red cell, fresh frozen plasma, cryoprecipitate, platelet
Metabolic	Metabolic acidosis (worst pH 6.87), hypoglycemia (glucose 1.0 mmol/l), hypocalcemia (0.63 mmol/l)	Dextrose and NaHCO3 infusion; calcium supplementation
Neurologic	Convulsion	Anticonvulsant
Hepatic	Deranged liver function with worst total bilirubin of 125 umol/l and alanine aminotransferase 574 IU/l	Supportive and treating underlying infection

## Discussion

This report illustrates that PF is an ominous cutaneous sign of fulminant neonatal GBS septicemia. Childhood PF is often associated with meningococcaemia [1-3]. In the neonatal period group B streptococcus is the major cause of PF but gram negative organisms such as *Escherichia coli *and *Enterobacter *have been described [7-11].

Nolan et al reported two cases of PF associated with meningococcal and chickenpox, respectively, and reviewed various treatment modalities [1]. Protein C and antithrombin III have been given if these factors are deficient [1,5]. In PF associated with meningococcemia and septic shock, Rivard et al described severe acquired protein C deficiency successfully treated with conventional therapy and high-volume plasma exchange as a source of protein C [2]. Many other therapies have been described in case reports that claimed to arrest the progression of neonatal PF, such as the use of heparin [8], Protein C [5,12], Antithrombin III, recombinant tissue plasminogen activator (rtPA), epoprostenol (prostacyclin) [6], topical nitroglycerin, intravenous dextran, and plasmapheresis [1-13]. Nevertheless, there is no strong evidence in favor of one particular therapy due to the small number of cases.

One limitation of our case was that the coagulopathy was immediately treated empirically before the endogenous activities of the anticoagulant factors protein C, protein S, and Antithrombin III (AT III) were assessed. In case any of these factors are found to be affected, human protein C or recombinant human activated protein C may be considered, and protein C, protein S, and AT III genes should have been analyzed in the patient and her parents. There was no family history of coagulopathy and group B streptococcus was identified to be the pathogen, making syndromes of congenital anticoagulant factor deficiency unlikely in this patient.

Regardless of the pathogen, neonatal PF must be promptly recognized and aggressively treated in children [1-6]. Early goal-directed therapy provides significant benefits with respect to outcome in adult patients with severe sepsis and septic shock [14]. The therapy involves adjustments of cardiac preload, afterload, and contractility to balance oxygen delivery with oxygen demand. Guidelines were proposed through the Surviving Sepsis Campaign to improve outcome in septic patients [15]. They are difficult to apply routinely and validate in neonates. As Claessens et al has commented, attempts to apply all of the procedures recommended by the experts, despite the apparent pragmatism of those procedures, have varied widely; diagnosis may be problematic because of atypical or unspecific presentations, biomarkers are of little help at the start of treatment and are unspecific, supportive treatment often depends on local supply of resources, and specific devices are often absent for initial therapy and monitoring [15]. Resuscitation policy for septic shock in neonates generally includes prompt treatment of the underlying infection with broad spectrum antibiotics, replacement of fluids or blood for preload, appropriate usage of inotropes for cardiac contractility and afterload, support of oxygenation and ventilation with mechanical ventilation, and support of individual multi-organ system failure [16].

Despite full intensive care support, PF is often associated with multiple organ failure and high mortality in children [4]. PF has a reported mortality of 50 per cent secondary to multiple organ failure which commonly accompanies the syndrome and is associated with major long-term morbidity in those who survive [4]. In 3 cases of early neonatal PF, all the babies survived but had markedly compromised neurologic outcomes [7].

## Consent

Written informed consent was obtained from the patient's next of kin for publication of this case report and any accompanying images. A copy of the written consent is available for review by the Editor-in-Chief of this journal.

## Competing interests

The authors declare that they have no competing interests.

## Authors' contributions

All authors participated in the care of the patient. KH is the principal author who drafts the manuscript. KS, WW and KC ensure that the clinical data is accurate. WW provides dialysis care. All authors read and approved the final manuscript.
